# Pathogeny of Diabetes

**Published:** 1911-10

**Authors:** 


### Pathogeny of Diabetes

FRANK AND S. ISAAC, *Arch. für exp. Path. und Pharm.,* 1911, hold that the normal oxidation of carbohydrates is ultimately from glycogen, not glucose, and that pancreatic lesions prevent the liver and other organs from forming glycogen, on account of the lack of the internal secretion. They claim that, in diabetes, the liver and other organs, glands and muscles, are surcharged with glucose—as would be expected in accordance with any theory—and, especially that they contain only traces of glycogen. We have long felt that an understanding of diabetes required closer attention to the old-fashioned theory of glycogen formation. Still, if their contention that oxidation is ultimately from glycogen directly, without reconversion into dextrose is correct, how can we explain the normal content of dextrose in the blood, especially after administering levulose?

